# Effects of muscle fatigue on gait characteristics under single and dual-task conditions in young and older adults

**DOI:** 10.1186/1743-0003-7-56

**Published:** 2010-11-09

**Authors:** Urs Granacher, Irene Wolf, Anja Wehrle, Stephanie Bridenbaugh, Reto W Kressig

**Affiliations:** 1 Institute of Exercise and Health Sciences, University of Basel, Basel, Switzerland; 2 Institute of Sport Science, Friedrich-Schiller-University Jena, Jena, Germany; 3 Basel University Hospital, Division of Acute Geriatrics, Basel, Switzerland

## Abstract

**Background:**

Muscle fatigue and dual-task walking (e.g., concurrent performance of a cognitive interference (CI) while walking) represent major fall risk factors in young and older adults. Thus, the objectives of this study were to examine the effects of muscle fatigue on gait characteristics under single and dual-task conditions in young and older adults and to determine the impact of muscle fatigue on dual-task costs while walking.

**Methods:**

Thirty-two young (24.3 ± 1.4 yrs, *n *= 16) and old (71.9 ± 5.5 yrs, *n *= 16) healthy active adults participated in this study. Fatigue of the knee extensors/flexors was induced by isokinetic contractions. Subjects were tested pre and post fatigue, as well as after a 5 min rest. Tests included the assessment of gait velocity, stride length, and stride length variability during single (walking), and dual (CI+walking) task walking on an instrumented walkway. Dual-task costs while walking were additionally computed.

**Results:**

Fatigue resulted in significant decreases in single-task gait velocity and stride length in young adults, and in significant increases in dual-task gait velocity and stride length in older adults. Further, muscle fatigue did not affect dual-task costs during walking in young and older adults. Performance in the CI-task was improved in both age groups post-fatigue.

**Conclusions:**

Strategic and/or physiologic rationale may account for the observed differences in young and older adults. In terms of strategic rationale, older adults may walk faster with longer strides in order to overcome the feeling of fatigue-induced physical discomfort as quickly as possible. Alternatively, older adults may have learned how to compensate for age-related and/or fatigue-induced muscle deficits during walking by increasing muscle power of synergistic muscle groups (e.g., hip flexors). Further, a practice and/or learning effect may have occurred from pre to post testing. Physiologic rationale may comprise motor unit remodeling in old age resulting in larger proportions of type I fibres and thus higher fatigue-resistance and/or increased muscle spindle sensitivity following fatigue leading to improved forward propulsion of the body. These findings are preliminary and have to be confirmed by future studies.

## Background

The number of senior citizens aged 65 and older has substantially increased in societies of western industrial countries. A serious concern of these countries is that larger proportions of elderly people produce increased health expenditures and may thus undermine the sustainability of the public health care system [1]. A major reason for high medical treatment costs in older adults is an increased prevalence of sustaining falls and fall-related injuries [2]. Twenty-eight to 35% of individuals over the age of 65 years experience at least one fall over a one-year period [3] with 20% of falls requiring medical attention [4].

Gait instability in terms of greater stride-to-stride variability has been identified as a major intrinsic risk factor for falls in old age [5]. There is evidence that gait variability further deteriorates when two tasks (postural plus a secondary cognitive/motor task) are concurrently performed. In fact, Granacher et al. [6] found larger temporal and spatial stride-to-stride variability in older compared to young adults when walking under dual-task conditions (i.e., walking while reciting out loud serial subtractions by three) as compared to single-task conditions (i.e., only walking). Kressig et al. [5] suggested that the degree of stride time variability in dual-task walking conditions distinguished fallers from non-fallers in a group of independently walking, older inpatients. Further, a recent systematic review on dual-task performance and the prediction of falls indicated that changes in performance whilst dual-tasking were significantly associated with an increased risk for falling among older adults [7].

Recently, it was reported that decrements in postural control and thus the increased occurrence of falls are not only caused by biologic aging and dual-task interference, but also by fatigue of the lower leg muscles [8,9]. In fact, Parijat et al. [9] observed that localized muscle fatigue of the quadriceps affected various kinematic and kinetic gait parameters that are linked with a higher risk of slip-induced falls in young healthy adults. Helbostad et al. [8] reported that a repeated sit-to-stand task affected gait control in older persons in terms of an increased variability in step width and length. Yet, there are only few studies available in literature that investigated how the attentional demand associated with postural control is modified by muscle fatigue. Vuillerme et al. [10] observed that ankle fatigue induced an increase in attentional demand during the regulation of static postural control in young, healthy adults. However, to the authors' knowledge, there is no study available which investigated the impact of muscle fatigue on dynamic postural control under single and dual-task conditions in young and older adults. Thus, the objectives of this study were to examine the effects of knee extensor/flexor fatigue, as established by standard criteria, on gait characteristics under single and dual-task conditions in young and older adults and to find out the impact of muscle fatigue on dual-task costs while walking in these age groups. Based on the results of studies conducted by Granacher et al. [6], Helbostad et al. [8], and Vuillerme et al. [10], we hypothesized that localized muscle fatigue of the knee extensors and flexors results in greater gait instability (i.e., stride-to-stride variability) in young and older adults under single and dual-task conditions. Further, we expected increased dual-task costs while walking in the fatigued condition, particularly in the elderly.

## Methods

### Participants

Thirty-two healthy young (*n *= 16) and elderly (*n *= 16) community-dwelling participants gave written informed consent to participate in the study after experimental procedures were explained (Table 1). The participants were healthy with no previous lower extremity trauma and no history of serious muscular, neurological, cardiovascular, metabolic and inflammatory diseases. The elderly subjects were capable of walking independently without any assistive device and they had no prior experience with the applied tests. The study was approved by the ethics committee of the University of Basel and all experiments were conducted according to the latest revision of the declaration of Helsinki.

**Table 1:** Characteristics of the study cohort

Characteristic	Young adults (*n *= 16)	Older adults (*n *= 16)
Age (years)	24.3 ± 1.4	71.9 ± 5.5
Sex (f/m)	8/8	8/8
Body height (cm)	173.7 ± 8.6	171.1 ± 10.2
Body mass (kg)	67.1 ± 9.4	73.8 ± 10.1
Everyday and sports-related PA level (h/week)	12.0 ± 3.4	9.7 ± 4.4
TUG (s)	8.1 ± 1.2	9.5 ± 1.6
Right KE strength in unfatigued condition (psi)	207.5 ± 78.3	83.6 ± 44.3
Right KF strength in unfatigued condition (psi)	146.1 ± 45.4	62.3 ± 32.0
Left KE strength in unfatigued condition (psi)	215.4 ± 77.6	113.0 ± 26.6
Left KF strength in unfatigued condition (psi)	137.5 ± 35.2	78.2 ± 24.5
Repetitions to reach 50% of M_max_	41.6 ± 23.3	70.4 ± 27.5
RPE after the fatigue protocol	16.9 ± 1.5	16.1 ± 1.8

*Note: *Values are mean ± SD. f = female; m = male; PA = physical activity; TUG = timed up and go test; KE = knee extensor; KF = knee flexor; RPE = rate of perceived exertion

### Testing

Upon entering the gait laboratory, participants received instructions regarding the test procedure with a visual demonstration of the walking and the strength tests. Thereafter, subjects performed one practice trial under single and dual-task conditions on the pressure-sensitive walkway to rule out potential learning effects in the post and follow up tests. Further, the Timed Up & Go Test (abnormal mobility was defined as a time ≥ 20 s [11]) was conducted. In addition, participants were seated and fixed on the isokinetic device in order to become acquainted with the test apparatus. After having completed the acquisition phase, subjects were asked to answer the questions of three different questionnaire (Freiburg questionnaire for everyday and sports activities, Mini Mental State Examination (MMSE), Falls Efficacy Scale-International (FES-I)) and one cognitive test to evaluate executive function (Clock Drawing Test (CDT)). Thereafter, the initial gait analysis (unfatigued) was conducted under single and dual-task condition, followed by the isokinetic fatigue protocol. Subsequently, post (right after the fatigue protocol) and follow up (after a 5 min rest) gait analyses were executed to investigate the effects of muscle fatigue and the acute recovery from muscle fatigue on gait characteristics under single and dual-task conditions in young and older adults.

### Apparatus

#### Gait analysis

Test circumstances (e.g., room illumination, temperature, noise) were in accordance with recommendations for posturographic testing [12]. Measurements were carried out in our gait laboratory and included the assessment of gait characteristics while walking on a pressure sensitive 10-m walkway using GAITRite^®^-System (Havertown, USA). Participants walked with their own footwear at self-selected speeds, initiating and terminating each walk a minimum of 2 m before and after the 10-m walkway to allow sufficient distance to accelerate to and decelerate from a steady state of ambulation across the walkway. Distribution of pressure during walking was monitored at 80 Hz, enabling data collection of gait velocity, stride length, as well as spatial stride-to-stride variability. Because data from the left and right strides were not statistically different, only data from the left side were analyzed. Besser et al. [13] reported that 5 to 8 strides are necessary for 90% of individuals tested with GAITRite(r) instrumentation to have reliable mean estimates of spatiotemporal gait parameters. Given that gait variability is a marker of gait stability/instability and fall risk [14,15], spatial stride-to-stride variability was computed. Therefore, the coefficient of variation (CV) was calculated for stride length according to the following formula [(SD/Mean)*100] [14] and used as an outcome measure. The smaller the CV value, the better the walking pattern. In addition, gait velocity and stride length were analyzed. Intraclass correlation coefficients for our gait parameters ranged from ICC = .66 to. 86 for the different task conditions.

#### Cognitive interference task

Gait characteristics were also examined while performing a concurrent attention-demanding CI task. The CI task was an arithmetic task, in which the participants recited out loud serial subtractions by three starting from a randomly selected number between 300 and 900 given by the experimenter [16]. When dual-task methodology was used, participants were instructed to give equal priority to both tasks in order to create real life conditions [17]. A recently conducted study indicated that task prioritization had no effect on measures of postural control while dual-tasking [18]. All tests were performed in a counterbalanced order for single and dual-task conditions. Evaluation of the performance of the cognitive interference task was done by taking the total number of subtractions minus the number of subtraction mistakes made during the task [19]. The higher the total subtraction number, the better the performance. Dual-task performance of our subjects was additionally quantified by calculating dual-task costs for each subject and parameter according to the following formula [(single-task score - dual-task score)/single-task score)*100] [20].

#### Questionnaire

The "Freiburg questionnaire for everyday and sports activities(c)" [21] assesses basic physical activity level (e.g., gardening, climbing stairs), leasure time physical activity level (e.g., dancing, bowling), and sports activity level (e.g., jogging, swimming) of people between the ages of 18 to 78 years. Significant test-retest reliability was reported for the summed physical activity level (*r *= .56). Cross-correlation with maximum oxygen uptake revealed a significant correlation coefficient of *r *= .42 [21].

The Mini Mental State Examination (MMSE) was applied which is a valid screening test of cognitive function. It separates patients with cognitive disturbance from those without such disturbance. Test-retest reliability of the MMSE is high with *r *= .89. Cross-correlation with the "Wechsler Adult Intelligence Score" revealed a correlation coefficient of *r *= .78 [22]. According to Folstein et al. [22], a MMSE total score of less than 20 separates patients with dementia or functional psychosis from normal participants and those with anxiety neurosis or personality disorder.

The Clock Drawing Test (CDT) is a sensitive screening test for the evaluation of executive function [23]. The elderly participants were instructed to draw numbers in a given circle to make the circle look like a clock. Thereafter, subjects were asked to draw the hands of the clock to a specific point in time. Depending on the study consulted, interrater reliability for the CDT ranges between 75.4 to 99.6% [23]. Test-retest reliability can be classified as high with a *r*-value of .90 [24]. Cross-correlation with the MMSE revealed a correlation coefficient of *r *> .50 [25]. As a result, the test distinguishes between pathological patients and healthy individuals.

The Falls Efficacy Scale-International (FES-I) was developed for the documentation of fall-related self efficacy in older persons. The FES-I showed excellent internal and test-retest reliability (Cronbach's α = .96, intraclass correlation coefficient (ICC) = .96). In addition, the FES-I has been shown to have acceptable construct validity in different samples in different countries (range *r *= .79 to .82) [26].

### Isokinetic fatigue protocol

Bilateral fatigue was induced by performing repetitive isokinetic knee extension movements of the quadriceps. The fatigue inducement procedures were similar to those described by Yaggie and McGregor [27], with the exception that bilateral fatigue of the quadriceps was used. All exertions were performed at 60°/s a value consistent with an earlier fatigue protocol [28]. Right after the initial gait analysis, participants' shoulders, waist and thighs were firmly fixed in a seated position in the isokinetic device (Cybex(r) K2, Medway, USA). Before the protocol started, subjects became accustomed to the isokinetic device by doing a warm-up consisting of five submaximal dynamic actions in a concentric-concentric mode. Thereafter, each subject performed four maximal contractions of the knee-extensors and flexors at 60°/s. For each trial, subjects were thoroughly instructed to act as forcefully as possible. The best trial was taken as maximal torque (M_max_). The fatigue criteria were determined by examining the subjects' M_max _during each exercise. No limitations were placed on the number of repetitions to reach 50% of M_max_. During the fatigue protocol, subjects were instructed to avoid forced respiration during maximal efforts. Once three consecutive repetitions below 50% M_max _were obtained, subjects were asked to estimate rate of perceived exertion on a 6 to 20 Borg scale [29]. Thereafter, participants were unfixed from the isokinetic device and led to the instrumented walkway to perform walks under single and dual-task conditions in a fatigued state. In order to determine the ability to recover from muscle fatigue, walks were repeated 5 minutes (T5) after the fatigue protocol.

### Statistical analysis

Data are presented as group mean values ± standard deviations (SD). A multivariate analysis of variance (MANOVA) was used to detect differences between study groups in all baseline variables. The effects of muscle fatigue on gait parameters under single and dual-task conditions were analyzed in separate 2 (Groups: young, old) × 3 (Tests: pre, post, follow up) analysis of variance (ANOVA) with repeated measures on test. Further, our ANOVA model was corrected for baseline values of gait velocity, maximal torque of the knee extensors, and gender. Post hoc tests with the Bonferroni-adjusted α were conducted to identify the comparisons that were statistically significant. In addition, the classification of effect sizes (*f*) was determined by calculating partial eta square (*η^2^*_p_). The effect size is a measure of the effectiveness of a treatment and it helps to determine whether a statistically significant difference is a difference of practical concern. *f *- values = .10 indicate small, *f *- values = .25 medium, and *f *- values = .40 large effects [30]. An a priori power analysis [31] with an assumed Type I error of 0.05 and a Type II error rate of 0.20 (80% statistical power) was conducted for gait measurements [8] and revealed that 16 persons per group would be sufficient for finding statistically significant interaction effects. The significance level was set at *p *< .05. All analyses were performed using Statistical Package for Social Sciences (SPSS) version 17.0.

## Results

### Questionnaire

The investigated results in the MMSE (mean: 28.7 ± 1.1; range: 27-30), the CDT (all subjects were classified as non-pathological), and the FES-I (mean: 18.7 ± 2.7; range 16-24) indicate that the elderly participants of this study were cognitively healthy without any serious concern about falling. Findings regarding the "Freiburg questionnaire for everyday and sports activities(c)" reveal that our participants can be classified as physically active (Table 1). Further, no statistically significant differences in anthropometric measures (i.e., body height/mass) and in the level of physical activity were found between the two experimental groups.

### Isokinetic fatigue protocol

At baseline, significant differences between young and older adults were observed in terms of maximal torque of the right and left knee extensors and flexors (all *p *< .01). Further, young adults needed significantly less repetitions to reach 50% of M_max _than the older adults. Post-fatigue, rate of perceived exertion on a 6 to 20 point Borg scale was not significantly different between the experimental groups (Table 1).

### Gait analysis

Table 2 displays means and standard deviations for all analyzed gait parameters. Results of the Timed Up & Go Test indicate that our young and elderly subjects were not mobility restricted (Table 1). Significant baseline differences between the experimental groups were found for stride length variability under dual-task conditions only.

**Table 2:** Effects of muscle fatigue on gait characteristics under single and dual-task conditions in young and older adults

Parameter	Young adults (*n *= 16)			Older adults (*n *= 16)		
	Pre	Post	T5	Pre	Post	T5
						
Gait velocity under ST condition (cm/s)	126.3 ± 16.6	121.8 ± 14.7	125.9 ± 17.1	124.2 ± 14.9	127.7 ± 13.7	125.5 ± 14.0
Gait velocity under DT condition (cm/s)	113.8 ± 15.5	110.9 ± 15.9	113.9 ± 15.0	106.2 ± 18.4	116.5 ± 18.7	116.6 ± 19.0
DT costs in gait velocity (%)	9.6 ± 8.4	9.1 ± 5.9	9.3 ± 5.8	14.1 ± 11.4	8.7 ± 10.9	7.5 ± 8.2
Stride length under ST condition (cm)	136.6 ± 15.8	132.6 ± 13.2	135.9 ± 14.4	138.6 ± 9.9	139.3 ± 9.9	138.3 ± 9.1
Stride length under DT conditions (cm)	129.9 ± 13.8	126.9 ± 14.9	129.5 ± 12.9	128.2 ± 11.7	133.8 ± 13.1	132.8 ± 12.5
DT costs in stride length (%)	4.7 ± 4.6	4.4 ± 3.6	4.6 ± 3.2	7.2 ± 5.9	4.0 ± 6.7	4.1 ± 4.8
Stride length variability under ST condition (cm)	1.7 ± 0.9	1.9 ± 0.8	1.2 ± 0.4	2.0 ± 0.7	1.9 ± 1.0	1.8 ± 0.9
Stride length variability under DT conditions (cm)	1.9 ± 0.8	2.0 ± 0.4	1.9 ± 0.7	4.0 ± 2.8	2.0 ± 1.0	2.6 ± 0.9
DT costs in stride length variability (%)	-50.3 ± 95.9	-24.8 ± 71.8	-76.6 ± 109.7	-116.0 ± 177.1	-58.2 ± 145.1	-66.6 ± 87.1
Performance in the CI task during walking (number of correct subtractions)	6.1 ± 1.5	7.6 ± 1.4	7.4 ± 1.8	5.1 ± 2.0	6.0 ± 1.7	5.9 ± 1.5

*Notes: *Values are mean ± SD; ST = single task; DT = dual task; CI = cognitive interference

#### Gait velocity

**Figure 1:**
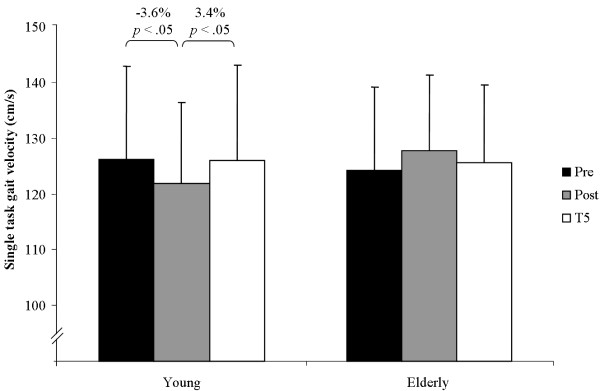
**Performance changes in gait velocity from pre, to post, to T5 testing under single-task conditions**.

**Figure 2:**
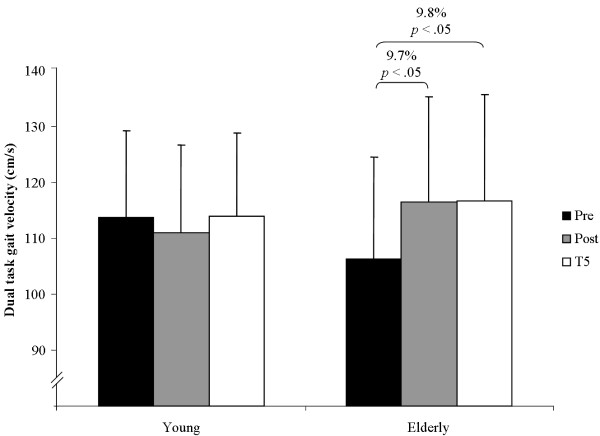
**Performance changes in gait velocity from pre, to post, to T5 testing under dual-task conditions**.

The statistical analysis indicated a significant main effect of test for gait velocity under dual-task conditions (*F*(2, 124) = 3.76, *p <*.05, *η*^2 ^= .111, *f *= .35) but not under single-task condition (*F*(2, 124) = 0.20, *p >*.05, *η*^2 ^= .007, *f *= .08). Main effects of group were not statistically significant for single-task (*F*(1, 30) = 0.26, *p >*.05, *η*^2 ^= .009, *f *= .10) and dual-task conditions (*F*(1, 30) = 0.09, *p >*.05, *η*^2 ^= .003, *f *= .05). Further, there were no significant main effects of test (*F*(2, 124) = 1.20, *p >*.05, *η*^2 ^= .040, *f *= .20) and group (*F*(1, 30) = 0.068, *p >*.05, *η*^2 ^= .003, *f *= .05) for the parameter dual-task costs in gait velocity. Group × Test interaction for dual-task costs in gait velocity showed a tendency towards significance (*F*(2, 124) = 2.70, *p *= .07, *η*^2 ^= .083, *f *= .30). Notably, Group × Test interaction for single-task (*F*(2, 124) = 3.31, *p <*.05, *η^2 ^*= .099, *f *= .33) and for dual-task gait velocity (*F*(2, 124) = 6.38, *p <*.01, *η^2 ^*= .175, *f *= .46) reached the level of significance. Post-hoc analysis revealed that young adults significantly decreased their gait velocity under single-task condition from pre to post testing and increased it again from post to T5 testing (Figure 1). In addition, older adults significantly increased their gait velocity under dual-task conditions from pre to post testing and from pre to T5 testing (Figure 2).

#### Stride length

**Figure 3:**
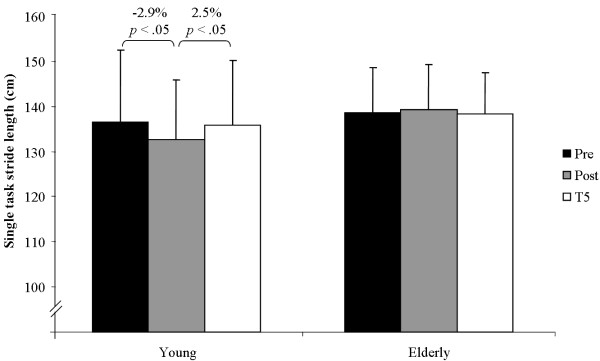
**Performance changes in stride length of the left leg from pre, to post, to T5 testing under single-task conditions**.

**Figure 4:**
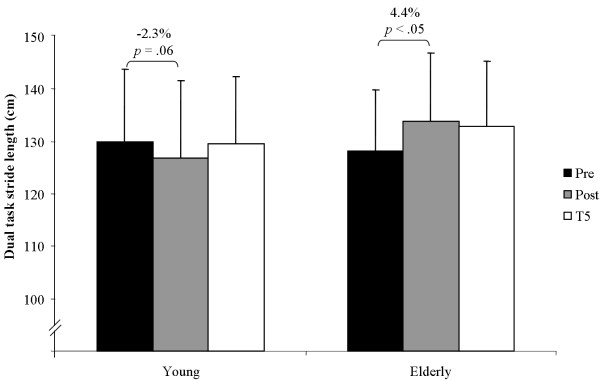
**Performance changes in stride length of the left leg from pre, to post, to T5 testing under dual-task conditions**.

The statistical analysis did not find significant main effects of test for stride length under single (*F*(2, 124) = 1.59, *p >*.05, *η^2 ^*= .050, *f *= .23) and dual-task conditions (*F*(2, 124) = 1.13, *p >*.05, *η^2 ^*= .036, *f *= .19) and of group under single (*F*(1, 30) = 0.88 *p >*.05, *η*^2 ^= .028, *f *= .17) and dual-task conditions (*F*(1, 30) = 0.51 *p >*.05, *η*^2 ^= .017, *f *= .13). Further, main effects of test (*F*(2, 124) = 2.38, *p >*.05, *η^2 ^*= .073, *f *= .28) and of group (*F*(1, 30) = 0.13 *p >*.05, *η*^2 ^= .004, *f *= .06) were not statistically significant for dual-task costs in stride length. Group × Test interaction for dual-task costs in stride length did not reach the level of significance (*F*(2, 124) = 1.85, *p >*.05, *η^2 ^*= .058, *f *= .25). Yet, Group × Test interaction was significant for stride length under single (*F*(2, 124) = 3.51, *p <*.05, *η^2 ^*= .105, *f *= .34) and dual-task conditions (*F*(2, 124) = 5.71, *p <*.01, *η^2 ^*= .160, *f *= .44). Results of the post-hoc analysis showed that young adults significantly decreased their stride length from pre to post testing under single (Figure 3) and dual-task conditions (Figure 4) and increased it again from post to T5 testing under single-task condition (Figure 3). Older adults significantly increased their stride length from pre to post testing under dual-task conditions (Figure 4).

#### Stride length variability

The statistical analysis detected statistically significant main effects of test (*F*(2, 124) = 3.41, *p *< .05, *η^2 ^*= .102, *f *= .34) and of group (*F*(1, 30) = 6.47 *p <*.05, *η*^2 ^= .178, *f *= .46) for stride length variability under dual-task conditions. Yet, main effects of test for stride length variability under single-task condition (*F*(2, 124) = 2.10, *p *> .05, *η^2 ^*= .065, *f *= .26) and of group (*F*(1, 30) = 1.49, *p >*.05, *η*^2 ^= .047, *f *= .22) were not significant. In addition, no significant main effects of test (*F*(2, 124) = 0.92, *p *> .05, *η^2 ^*= .030, *f *= .18) and of group (*F*(1, 30) = 1.89, *p >*.05, *η*^2 ^= .059, *f *= .25) were found for dual-task costs in stride length variability. Group × Test interactions for dual-task costs in stride length variability (*F*(2, 124) = 0.72, *p *> .05, *η^2 ^*= .023, *f *= .15) and for stride length variability under single-task condition (*F*(2, 124) = 1.48, *p *> .05, *η^2 ^*= .047, *f *= .22) were not statistically significant. However, a significant Group × Test interaction was observed for stride length variability under dual-task conditions (*F*(2, 124) = 4.53, *p *< .05, *η^2 ^*= .131, *f *= .39). Post-hoc analysis specified that older adults significantly decreased their stride length variability from pre to post testing (Table 2).

Controlling our statistical analyses for gait velocity and/or gender did not affect conclusions of the statistical tests. However, when adjusting for maximal torque of the knee extensors, initially significant Group × Test interaction effects for gait velocity, stride length, and stride length variability were no longer present.

### Cognitive interference task

Significant main effects of test (*F*(2, 124) = 11.49, *p *< .01, *η^2 ^*= .277, *f *= .62) and of group (*F*(1, 30) = 7.86, *p <*.01, *η*^2 ^= .208, *f *= .51) were observed for the parameter performance in the cognitive interference task while walking. However, Group × Test interaction did not reach the level of significance (*F*(2, 124) = 1.02, *p *> .05, *η^2 ^*= .033, *f *= .18).

## Discussion

The study examined the effects of localized muscle fatigue on gait velocity, stride length, and stride length variability under single and dual-task conditions in young and older adults. The main findings can be summarized as follows. First, significantly lower maximal torque of the knee extensors and flexors were observed at baseline in older compared to young adults. Second, older adults needed significantly more repetitions to reach 50% of M_max _of the knee extensors/flexors than the young adults. Third, stride length variability under dual-task conditions was significantly greater at baseline in older compared to young adults. Fourth, localized muscle fatigue resulted in significant decreases in single-task gait velocity and stride length in young adults. Fifth, muscle fatigue produced significant increases in dual-task gait velocity and stride length in older adults which were accompanied by significant decreases in stride length variability under dual-task conditions. Sixth, muscle fatigue did not significantly affect dual-task costs in all analyzed gait parameters in both, young and older adults. Finally, muscle fatigue resulted in significant improvements in cognitive performance during walking in young and older adults. These findings indicate that our initially formulated hypothesis (i.e., localized muscle fatigue affects gait characteristics in young and older adults under single and dual-task conditions, and increases dual-task costs while walking, particularly in the elderly) is only partially supported.

### Differences in maximal torque between young and older adults

In this study, significant baseline differences between young and older adults were found for maximal torque of the knee extensors and flexors. This is consistent with the literature because Vandervoort et al. [32] examined a 53% lower concentric peak torque of the knee extensors in old as compared to young adults. The observed difference in maximal torque in this study could be caused by a reduced excitability of efferent corticospinal pathways resulting in lower levels of central muscle activation, a gradual loss of spinal motoneurons (particularly large alpha-motoneurons) due to apoptosis, a subsequent decline in muscle fibre number and size (sarcopenia) of especially type-II fibres, changes in muscle architecture, and decreases in tendon stiffness. For a review see Granacher et al. [33].However, due to the methodological approach applied in this study, we cannot directly infer on the underlying neuromuscular mechanisms responsible for the reduced level of maximal torque in old compared to young adults.

### Differences in fatigue-resistance between young and older adults

Findings of this study indicated that the older adults needed significantly more repetitions to reach 50% of M_max _of the knee extensors/flexors than the young adults. This may suggest that the older adults were more fatigue resistant than the young adults. In fact, there is evidence showing that older adults fatigue less than young during isometric contractions [34]. This can be explained by the occurrence of motor unit remodeling in old age, i.e., type II muscle fibres are denervated due to degenerative processes and subsequently re-innervated by an adjacent slow-twitch motor neuron resulting in a muscle fibre shift from type II to fatigue resistant type I fibres [35]. Thus, proportionally more fatigue-resistant type I fibres contribute to force generation in the aged compared to the young muscle. This may explain why the older adults needed significantly more repetitions to reach 50% of M_max _of the knee extensors/flexors than the young adults.

### Differences in stride length variability between young and older adults

The observed baseline differences in stride length variability under dual-task conditions between the two experimental groups are in accordance with a study conducted by Granacher et al. [6]. These authors reported increased stride length variability in old compared to young subjects when walking while concurrently performing a cognitive (i.e., performing an arithmetic task) or a motor interference task (i.e., holding two interlocked sticks steady in front of the body). Gunning-Dixon and Raz [36] attributed age-related dual-task deficits to the shrinkage of prefrontal brain areas in old age, since those areas are related to executive functions (e.g., processing of multi-tasking). Of note, it was recently shown in healthy older adults that individuals with poorer executive function are more prone to falls [37]. Other authors ascribe increased gait instability in old age to the age-related loss of visual, proprioceptive, and vestibular sensitivity [38]. Notably, baseline differences between the experimental groups were only present for stride length variability under dual-task conditions, but not for the parameters stride length and gait velocity under single and dual-task conditions. This contradicts findings reported by Hollman et al. [39] and Hausdorff et al. [40] who observed differences between young and older adults in gait velocity as well as in variability of stride time, stance time, and swing time. The lack of age-related effects on gait velocity and stride length observed in this study can quite likely be explained by the high physical activity level of our older subjects which was not significantly different from that of the young adults. In addition, subjects in the studies of Hollman et al. [39] and Hausdorff et al. [40] were older than our subjects with a mean age of 81 and 82 years respectively, which might explain why their gait pattern was characterized by greater instability.

### Effects of muscle fatigue on gait characteristics in young and older adults

The present results regarding the effects of muscle fatigue on gait characteristics in young adults are consistent with findings reported by Parijat et al. [41]. These authors examined the impact of bilateral fatigue induced by repetitive isokinetic knee extension movements of the quadriceps on kinematic and kinetic gait characteristics in healthy young adults. After fatigue exertions, participants showed a tendency towards a decrease in gait speed. It was argued that reduced push-off force during the stance phase of the gait cycle reduces the transitional acceleration of the whole body centre of mass and may thus be responsible for the decrease in gait velocity in young adults [41]. Reduced gait speed may represent a compensatory strategy to enhance dynamic stability during walking in order to keep from falling [41].

In our older adults, muscle fatigue produced an increase in gait speed and stride length coming along with a decrease in stride length variability particularly under dual-task conditions. This is in accordance with findings from two studies [42,43]. Morris et al. [42] investigated changes in gait characteristics (tested on a walkway) and fatigue from morning to afternoon in people with multiple sclerosis. Although self rated fatigue significantly increased from the morning to the afternoon, increases in walking speed and stride length were observed over the course of the day. The authors suggested that practice effects could be responsible for the observed increases over the course of the trials. Yoshino et al. [43] examined how long-term free walking (3 hours) at a self-determined preferred pace on level ground affected the gait pattern of healthy subjects. Based on their level of performance during the 3 h walk, subjects were assigned to two groups. Group A showed longer gait cycle time during the second half of the walk and group B showed shorter gait cycle time during the same period. Variability of the parameter gait cycle time increased significantly in group A from 120 min on, whereas it tended to decrease gradually with time in Group B. For both groups, the mean subjective levels of fatigue increased monotonically with time. The mean heart rate during the walking task was almost constant until 120 min from the beginning, and it tended to increase gradually during the last 60 min in both groups. Unfortunately, the authors did not provide a reasonable explanation for this phenomenon. It was suggested that subjects in group B could have been more fatigue resistant than those in group A because of higher levels of stamina [43].

Four reasons may account for the observed fatigue-induced increase in gait speed and stride length in the older adults. First, walking faster with longer strides could represent a strategy of the older adults to overcome the short walking distance (10 m) and thus the feeling of physical discomfort due to muscle fatigue as quickly as possible. Therefore, it is suggested that future studies investigate the effects of muscle fatigue on gait characteristics by incorporating longer walking distances. In fact, longer distances may prevent older subjects from initially increasing their walking speed to levels higher than their preferred non-fatigued walking speed because they might not be able to keep up this walking speed for the entire distance. Second, it was reported that the age-related loss of ankle plantar flexor strength resulted in lower ankle plantar flexor power during the late stance phase of the gait. Interestingly, older adults learned to compensate for this muscular deficit by increasing hip flexor power [44]. It is proposed that our older adults may have compensated fatigue-induced decreases in knee extensors/flexors by increasing hip flexor power during walking. In contrast, young adults probably never learned this compensatory strategy due to a lack of need. Third, it was reported that muscle fatigue has an impact on muscle spindle function in terms of an increase in sensitivity of this mechanoreceptor [45]. Increased muscle spindle sensitivity may represent a fatigue-induced compensatory mechanism to maintain function and force output [46]. Given that muscle spindle sensitivity decreases in seniors due to increased spindle capsule thickness and a loss of intrafusal- and nuclear chain fibers [47], it is speculated that particularly older adults could benefit from this compensatory mechanism in terms of enhanced leg extensor muscle activation and thus improved forward propulsion of the body. This hypothesis needs to be proven in future studies. Fourth, a practice and/or learning effect from pre to post tests could have resulted in an increase in gait speed and stride length in the older adults. However, due to the standardized testing procedures and because improvements were only present from pre to post but not from post to T5 testing, it is postulated that practice/learning may only play a minor role.

The observed increase in gait velocity and stride length post-fatigue was accompanied by a decrease in stride length variability indicating improved gait stability. Yet, it was recently reported that stride-to-stride variability appears to be speed dependent [48]. Jordan et al. [48] observed that gait cycle variability was lowest at 100% and 110% of the preferred walking speed. Post-fatigue, our older adults showed a 2.8% and a 9.7% increase in gait speed under single-task and dual-task conditions as compared to the respective non-fatigued preferred walking speed. Both percentage rates are within the range of lowest gait cycle variability stated by Jordan et al. [48].

### Age-related effects of muscle fatigue on gait characteristics are task dependent

Recently, Granacher et al. [49] investigated the effects of ankle fatigue on the ability to compensate for decelerating gait perturbations during walking on a treadmill in healthy young and older adults. The authors reported that muscle fatigue affected the compensatory mechanisms of young and older adults in terms of significant decreases in reflex activity and increases in antagonist co-activity of lower extremity muscles. Since young and elderly subjects were affected to a similar extent by muscle fatigue, the authors proposed that age-related deteriorations in the postural control system did not specifically affect the ability to compensate for gait perturbations under fatigued condition [49]. However, the fatigue-induced changes in reflex activity may put young and older adults at high risk of sustaining a fall when encountering a balance threatening situation in a fatigued state. The finding that young and older adults showed similar fatigue-induced responses when compensating for gait perturbations contradicts the present results. In this study, muscle fatigue produced different gait characteristics in young and older adults. More specifically, young adults decreased their gait velocity and stride length particularly under single-task condition, whereas older adults increased their walking speed and stride length predominantly under dual-task conditions. In addition, young adults slightly increased their stride length variability under dual-task conditions, whereas older adults significantly decreased theirs. The observed discrepancy between the study of Granacher et al. [49] and ours can most likely be explained by different test conditions. Whereas Granacher et al. [49] investigated the impact of muscle fatigue on postural reflexes in young and older adults, we studied the effects of muscle fatigue on the gait pattern which is regulated by a complex interaction of reflexive and voluntary contributions to muscle activation [50]. Notably, it was reported that neural control of volitional limb movements differs in some fundamental ways in comparison to reactions that are evoked by postural perturbation [51].

### Effects of muscle fatigue on dual-task costs while walking in young and older adults

In the present study, muscle fatigue did not have a significant impact on dual-task costs in all analyzed gait parameters in both, young and older adults. Bock et al. [52] found that the occurrence of dual-task costs while walking in healthy young and elderly persons is task dependent with complex secondary tasks affording higher dual-task costs. Thus, the choice of our secondary task (reciting out loud serial subtractions by three) may have influenced the outcome of this study. Further, Simoneau et al. [53] investigated how moderate fatigue induced by fast walking on a treadmill challenged dynamic balance control in young healthy adults and whether the attentional demands for the performance of the balance task varied with fatigue. Fatigue induced an initial negative impact on balance control followed by a subsequent improvement in the performance of the balance task. Subjects achieved this performance enhancement by allocating a greater portion of the cognitive resources to the balance control task. In general, this finding seems to be in accordance with the results of the present study regarding the young adults. More specifically, our young participants chose a different strategy of allocating central resources than those in the study of Simoneau et al. [53] because we detected impaired performance in balance control following fatigue accompanied by improved performance in the cognitive interference task while walking. In other words, the young adults achieved better cognitive performance post-fatigue at the cost of impaired balance control.

Improvements in cognitive performance following fatigue were also observed in the older adults participating in this study. Emery et al. [54] evaluated the acute effects of exercise (i.e., 20 min bicycle ergometry stress test) on cognitive performance in a community-based sample of patients (mean age 68 ± 7 years) with chronic obstructive pulmonary disease (COPD). Acute exercise was associated with improved performance on the verbal fluency test, a measure of verbal processing. The fatigue protocol applied in the present study represents some kind of an acute bout of exercise and our results may thus be comparable to those investigated by Emery et al. [54]. It was suggested that improved neurotransmitter functioning in the brain could be responsible for the enhanced cognitive function following acute bouts of exercise or fatigue [54].

## Conclusions

Overall, the present study indicates that a muscle fatigue protocol with standardized fatigue criteria produces predominately age-specific effects on gait characteristics under single and dual-task conditions in young and older adults. In young adults, muscle fatigue resulted in significant decreases in single-task gait velocity and stride length, whereas in older adults, it produced significant increases in dual-task gait velocity and stride length. Strategic and/or physiologic rationale may account for the observed differences in young and older adults. In terms of strategic rationale, older adults may walk faster with longer strides in order to overcome the short walking distance and thus the feeling of fatigue-induced physical discomfort as quickly as possible. Alternatively, older adults may have learned how to compensate for age-related and/or fatigue-induced muscle deficits during walking by increasing muscle power of synergistic muscle groups (e.g., hip flexors). Further, a practice and/or learning effect may have occurred from pre to post testing. Physiologic rationale may comprise motor unit remodeling in old age resulting in larger proportions of type I fibres and thus higher fatigue-resistance and/or increased muscle spindle sensitivity following fatigue leading to improved forward propulsion of the body. These findings are preliminary and have to be confirmed by future studies.

## List of abbreviations

CI: cognitive interference; CV: coefficient of variation; FES-I: Falls efficacy scale-international; ICC: intraclass correlation coefficient; M_max_: maximal torque; MMSE: Mini mental state examination; *η^2^*_p_: partial eta square; SD: standard deviation; T5: third test, five minutes after the post test;

## Competing interests

The authors declare that they have no competing interests.

## Authors' contributions

UG developed the study design, assisted in the recruitment of subjects, managed data acquisition, evaluated the data, performed data analyses, and wrote the manuscript. IW participated in the design of the study, recruited subjects, did the testing, participated in data analysis and drafting of the manuscript. AW participated in the design of the study, assisted in the recruitment of subjects, did the testing, participated in data analysis and drafting of the manuscript. SB participated in the design of the study, assisted in the recruitment of subjects, did the testing, participated in data analysis and drafting of the manuscript. RWK developed the study design, assisted in the recruitment of subjects, managed data acquisition and drafting of the manuscript. All authors read and approved the final manuscript.
